# The management of the bilateral internal carotid dissection clinical case presentation

**Published:** 2012

**Authors:** Ş Dima, C Scheau, F Ştefănescu, S Tuţa

**Affiliations:** * Department of Cerebral Angiography, National Institute of Neurology and Cerebrovascular Diseases in Bucharest; Carol Davila University of Medicine and Pharmacy, Bucharest, Romania; ** Department of Radiology and Medical Imaging, “Fundeni” Clinical Institute, Bucharest, Romania; “Carol Davila” University of Medicine and Pharmacy, Bucharest, Romania; *** Neurosurgery Clinic, National Institute of Neurology and Cerebrovascular Diseases from Bucharest; Carol Davila University of Medicine and Pharmacy, Bucharest, Romania; **** Neurology Clinic, National Institute of Neurology and Cerebrovascular Diseases from Bucharest; Carol Davila University of Medicine and Pharmacy, Bucharest, Romania

**Keywords:** Bilateral carotid dissection, spontaneous, carotid stenting, Wallstent, iatrogenic dissection, Magnetic Resonance Imaging (MRI), Computer Tomography (CT).

## Abstract

**Introduction:**We present the case of a 36-year-old patient who was treated in the National Institute of Neurology and Cerebrovascular Diseases in Bucharest – in the neurology and the imagistic departments - for bilateral carotid dissection.

**Goals:** The main goal of this article was to discover the cause that lead to the symptoms of the patient using MRI and angio-MRI.In the process,we tried to dilate the stenosis (due to dissection) on 2 internal carotid arteries by using stents in order to keep the true lumen open.

**Methods:**In order to make a diagnosis we used the Magnetic resonance imaging machine (MRI) (1,5 T from GE), the multislice Computer Tomography (CT) scan (16 detectors from Siemens) and the digital substraction angiography (Siemens Axiom Artis). In addition, we used the same angiography machine for the endovascular procedure. The stents that we used were Wallstents from Boston Scientific Company.

**Results:** The patient left the hospital having a NIHSS=10, with dysarthria and left hemiplegia that were 80% recovered after 2 months.

**Discussion:** The particularity of this case study is the spontaneous bilateral internal carotid dissection. The second dissection might have resulted in being also iatrogenic, due to several attempts of stenting the first one.

**Conclusions:** The successful treatment of this patient was the result of the collaboration between the neurology and neuroradiology departments.The first therapeutic option in carotid dissection has to be stenting, under certain conditions.

## Introduction:

We present the case of a 36-year-old womanwho had as previous symptoms: headaches and increased blood pressure during pregnancy thatlasted even after birth, for 3 years. She neglected the treatment but she was taking estroprogestativecontraceptive pills.

Clinical features:

The patient came to the hospital on 02.09.2011, with sensitive and transitory motor dysfunction on the upper left arm andwith a headache that lasted for 3-4 hours remitting spontaneously. The neurologic examination turned to be normal, but the blood pressure was of 160/100 mmHg. She was sent home with a prescription of amlodipinethat should have been taken orally.

On 02.11.2011, at 16:25 she had sudden left hemiplegia and dysarthria. She called the ambulance and at 19:00, she arrived in the emergency room of our hospital. The neurologic examination revealed left neglected hemiplegia, left hypoestesis and Claude Bernard Horner syndrome on the right side of the body scoring NIHSS:18, Glasgow:15, and TA=160/100mm Hg both arms.

Imaging diagnostic:

We performed an emergency MRI that showed a hyperintensity on the diffusion weightedsequence located in the right medium cerebral artery territory, without a correspondenton the FLAIR sequence,suggesting that the patient had a stroke recently. We also found hyperintensity images both on diffusion and on FLAIR in the territory of the angular artery and posterior parietal artery on the right side, responsible for the event that occurred on the 02.09.2011.

**Fig.1:**
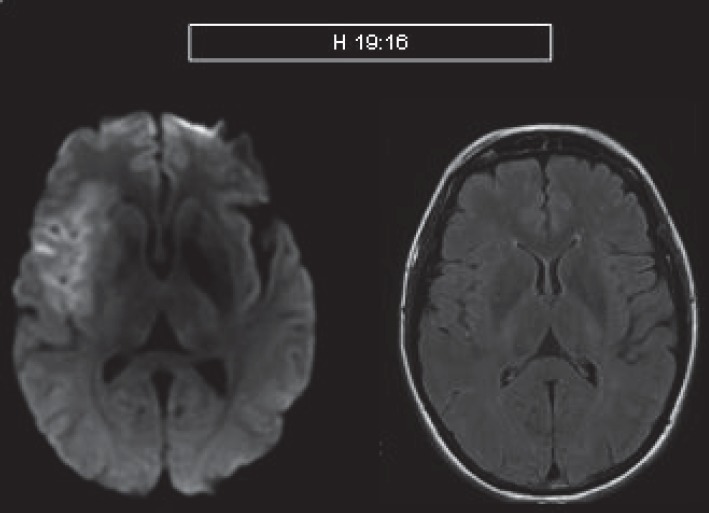
MRI diffusion with hyperintensity(left) – overacute ischemic lesion versus normal FLAIR sequence (right).

**Fig.2:**
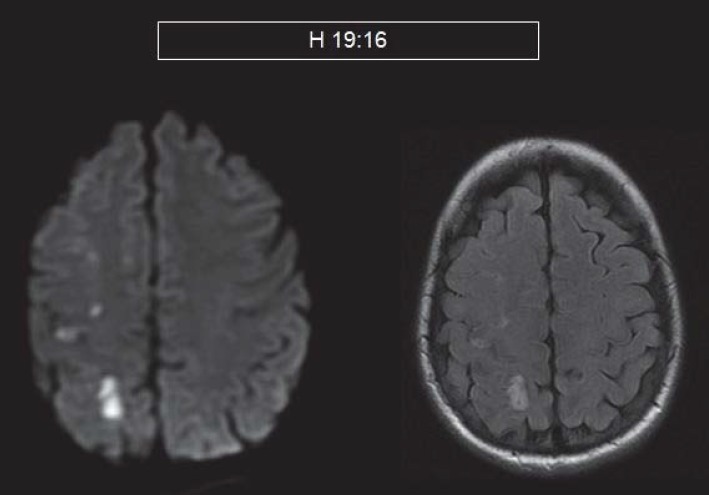
MRI- diffusion (left) and FLAIR hyperintensities (right) that suggests that there are irreversible ischemic lesions

Angio-MRI and axial T2 and FAT-SAT sequences suggest a post bulbar dissection of the right ICA and a normal left ICA.

**Fig.3:**
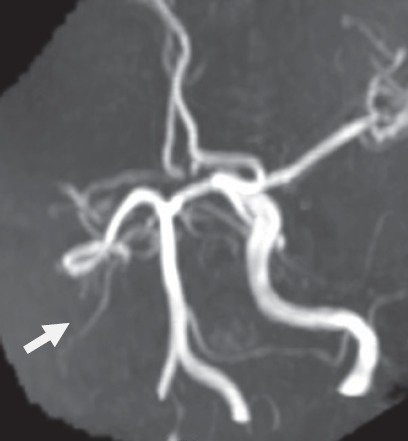
Angio-MRI no flow in the right ICA

**Fig. 4:**
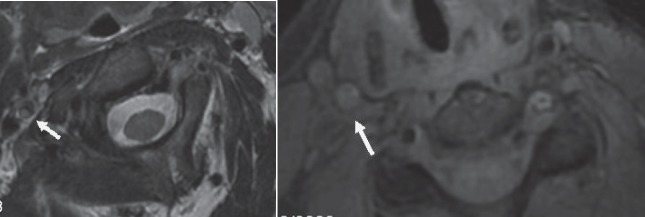
MRIT2 (left) and FAT-SAT (right) sequences reveal the dissection

The diagnostic consisted in right sylvian acute stroke and some lesions of the right sylvian subacute stroke and on the right ICA occlusion.

The treatment:

The first option that we considered was i.v. thrombolysis[**1-5**].

Arguments for it:

• therapeutic window (below 3 hours)
• right sylvian acute stroke

Arguments against it:

• sylvian subacute stroke from 02.09.2011

Another option that we considered was the endovascular treatment with passing the lesion and putting a stent intothe place. The entire team agreed that an angiography of 20.30 I was required.

**Fig. 5 a,b:**
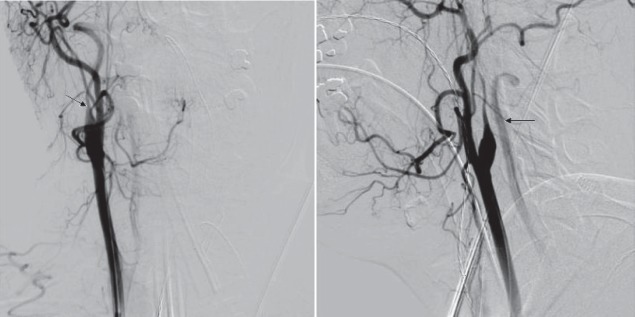
Digital substraction angiography – right ICA dissection

We confirmed the right ICA dissection and we also tried several times to find the true lumen.Unfortunately,each time, we entered only into the fake channel.

**Fig. 6 a,b:**
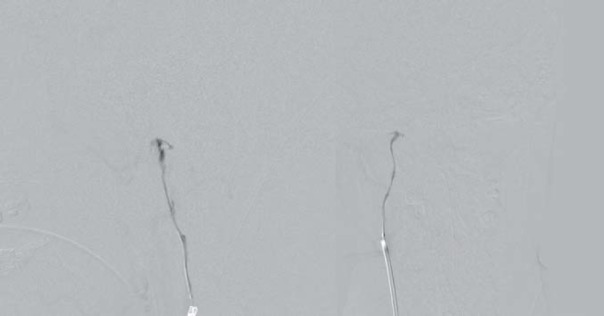
Digital substraction angiography – the fake chanel

Then we tried to pass from the left ICA via anterior communicating artery to the right ICA or from the posterior circulation via posterior communicating artery but we did not succeed. After 90 minutes of several atempts we did not manage to dilate the true chanel in order to put a stent inside.

**Fig. 7 a,b:**
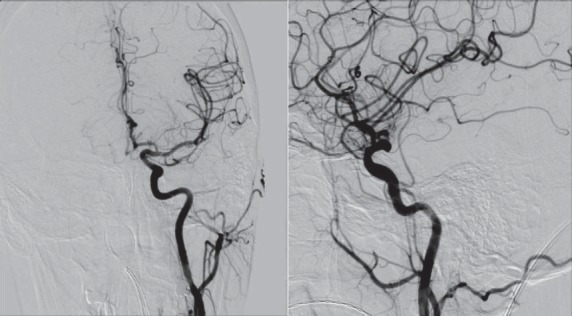
Digital substraction angiography – left ICA

Afterwards, we started the treatment with aspirin 250 mg p.o./day [**2**] and we tried to make an etiologic check of the stroke. It was not the typical case for fibrodisplasia, no traumatic marks, no hyperlaxity of the ligaments.

Patient’s investigations and follow-up:

The blood test and cardiology- were normal. The angio-CT for the renal arteries- was normal.

**Fig. 8:**
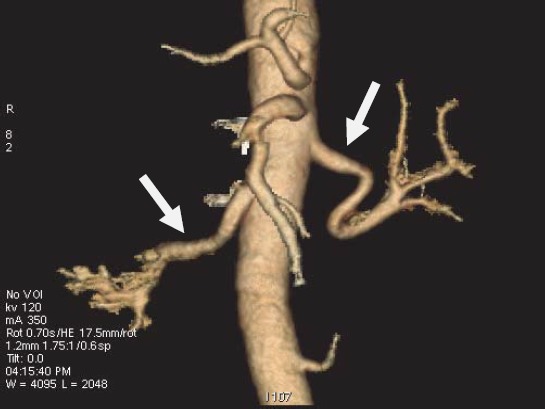
Angio-CT for the renal arteries.

Preliminary conclusion:essential arterial hypertension.

**Follow-up of the dissection:**

02.14 - Ultrasound Doppler for the supraaortic vessels showed ICA dissection and a 2-3mm fake channel and a parietal hematoma. Transcranian Doppler – slow flow on the right MCA.

02.20- The same dissection

02.22 -The patient had a moment of aphasia and comprehensive problems that lastedfor only 5 min. We immediately practiced a cerebral angio-CT and we discovered a dissection of the left ICA this time.

**Fig.9:**
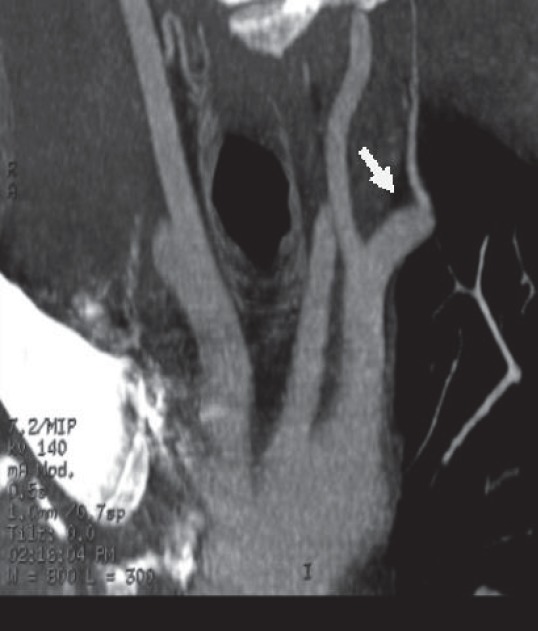
Angio-CT left ICA dissection

Only few moments after the angio-CT the patient had a second episode of aphasia and also hemiplegia of the right upper arm.

We performed an emergency MRI in the region of the left MCA.

**Fig. 10:**
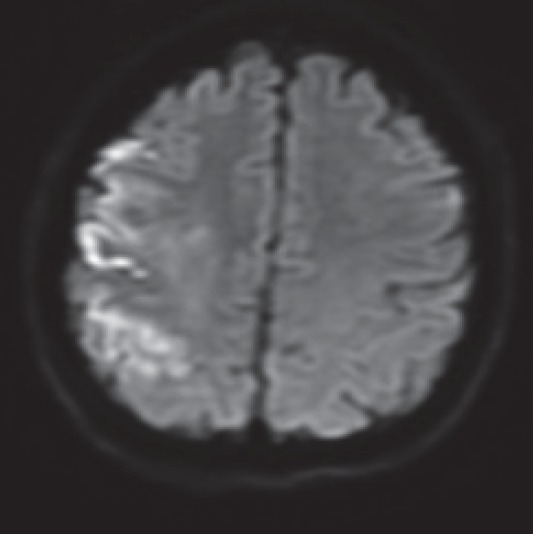
MRI diffusion sequence with right cortical fronto-parietal hyperintensity (the already known ischemic lesions), but also small left frontal new lesions

We decided to try the endovascular treatment as soon as possible. This time we managed to deploy 2 carotid stents for opening the entire dissection.

**Fig.11 a,b:**
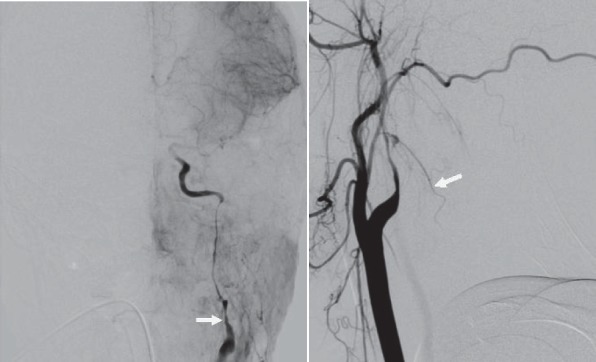
Digital substraction angiography- left ICA with dissection

**Fig.12 a,b:**
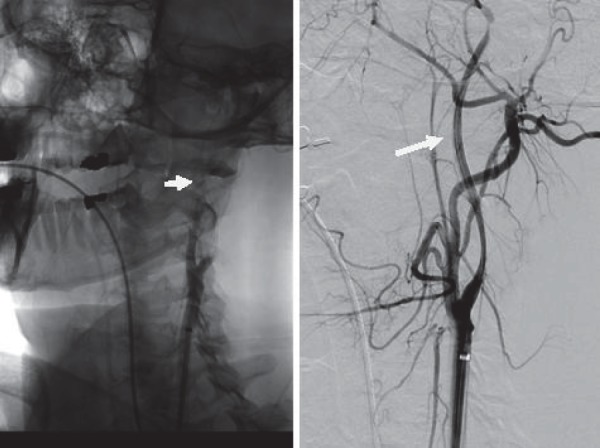
Digital angiography without substraction and with substraction after the first stent

**Fig.13 a,b:**
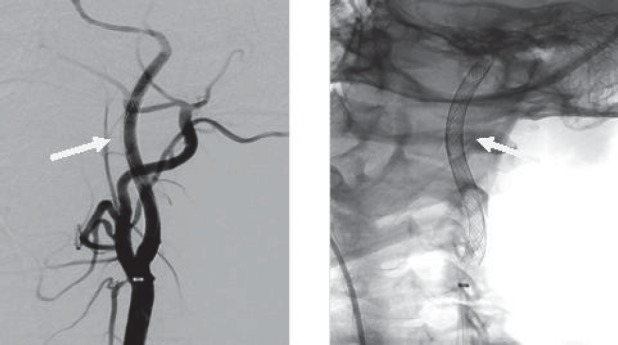
Digital angiography with substraction(a) and without substraction(b) after the second stent

**Fig. 14 a,b:**
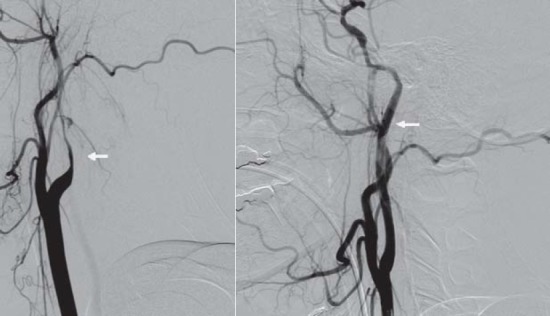
Digital substraction angiography before (a) and after stent (b)

The evolution was positive after the endovascular treatment and the patient fully recovered from the aphasia and had the right motor dysfunction but the left hemiplegia remained stable.

MRI after the endovascular treatment revealed a normal left cerebral hemisphere and the left ICA a normal flow. The Doppler from 03.04 showed also a perfect permeability of the stents and normal speed of the blood through them.

The patient left the hospital having a NIHSS=10, with dysarthria and still some left hemiplegia that were 80% recovered after 2 months.

## Discussion:

The particularity of this case is the spontaneous bilateral internal carotid dissection. The second dissection could have turned out to be iatrogenic, due to several attempts of stenting for the first one[**6-10**].

## Conclusions:

1) The successful treatment of this patient was a consequence of the collaboration between the neuroradiology and neurology departments [**11**].

2) The first therapeutic choice in carotid dissection has to be stenting as long[**12**].
